# A mixed methods exploration of the origin of dental anxiety and coping strategies among participants in a behavioral intervention for dental anxiety

**DOI:** 10.3389/froh.2025.1589764

**Published:** 2025-06-12

**Authors:** Elizabeth Konneker, Devon Singh, Marisol Tellez, Amid I. Ismail, Eugene M. Dunne

**Affiliations:** Department of Oral Health Sciences, Temple University Maurice H. Kornberg School of Dentistry, Philadelphia, PA, United States

**Keywords:** dental anxiety, dental care, qualitative research, coping skills, phobic disorders

## Abstract

**Introduction:**

Little is known about the onset and early progression of dental anxiety. The current mixed-methods study aimed to evaluate patient-reported early experiences and onset of dental anxiety, as well as the experience of managing dental anxiety (i.e., coping strategies and symptom severity).

**Methods:**

Adults (N=499) were recruited from a dental school clinic to participate in a clinical trial testing the efficacy of a cognitive behavioral therapy (CBT)-based intervention for dental anxiety. As one aspect of this trial, participants answered the Anxiety and Related Disorders Interview Schedule. During this interview, participants described when they first noticed developing anxiety about dental appointments, as well as how they coped during their appointments. Assessments were repeated at one-month and three-month follow-ups. Bivariate associations (e.g., chi-square and t-test) and repeated measures ANOVA were explored. Qualitative data were coded in NVivo.

**Results:**

The three identified origins for dental anxiety were: “traumatic dental visit in childhood,” “traumatic dental visit in adulthood,” and “anxiety has always been present.” Participants who reported a childhood trauma had the highest levels of dental anxiety relative to the other two groups. In total, 30 unique coping mechanisms were identified. A reduction in avoidant coping strategies was observed among the intervention groups at both one-month and three-months, but not in the control group.

**Discussion:**

Earlier negative dental experiences are more likely to result in greater anxiety severity. A one-time CBT-based dental anxiety treatment reduced the use of avoidant coping strategies, which may in turn reduce patient fears.

## Introduction

Dental anxiety, or the distress caused by anxiety about dental care, is a common occurrence among dental patients (1–3). In the United States, the prevalence of dental anxiety among dental patients is typically reported to be around 19% for adults (1) and 24% for children and adolescents (2). Worldwide, estimates of dental anxiety range from 12% to 15%, with slightly higher rates of dental anxiety among women and young adults (4, 5). One study of dental anxiety in Saudia Arabia reported an incidence of 51.6%, with 12.4% of participants reaching the threshold of extreme anxiety as measured by the Modified Dental Anxiety Scale (MDAS) (6).

Dental anxiety may meet diagnostic criteria for dental phobia if patients have been avoiding dental appointments specifically due to the anxiety they feel. This avoidance increases the likelihood of needing emergency care. Among adult patients seeking emergency care, prevalence rates of dental anxiety are estimated to be about 49% (3). Emergency visits may indicate postponement of care because of anxiety, are likely to involve more invasive procedures and are initiated as a result of significant dental pain. Dental anxiety in its severe form may also exacerbate preexisting negative feelings toward dental care (7).

While it is well understood that dental avoidance is likely to increase patient distress, there is limited information as to how dental anxiety initially develops (8). However, previous research on this topic has yielded promising findings that can improve clinicians’ understanding of the patient experience. In one study, the vividness of a mental picture related to dental anxiety, also known as being able to clearly imagine the images and sensations one might experience at an appointment, was associated with both a past distressing dental experience and high levels of dental anxiety, suggesting that patients’ vivid recollection of a painful dental visit may exacerbate future dental anxiety (9). The rate of dental anxiety also increases when patients require surgical, periodontal, or endodontic treatments (10).

A patient's coping ability may also influence their likelihood of avoiding dental appointments. However, coping with anxiety is not well understood because of the high levels of variation in coping strategies across individuals. Several existing studies have identified a positive relationship with one's dentist as a key factor for attending future dental appointments (11, 12), but this may reflect the interpersonal skills of the dentist more than a specific coping strategy used by patients during dental treatment. Optimism and optimistic thinking have also been identified as protective factors against dental avoidance (13). Research on coping strategies that may be maladaptive or ineffective is limited, though one study reported that patients with higher levels of dental anxiety tended to use avoidance or distraction-based coping behaviors. These behaviors include not attending a dental appointment or trying to mentally disengage from the dental appointment through a variety of methods (9).

The present study aimed to understand both the origins of dental anxiety and the coping strategies used by patients at a dental school in the US. Given the diversity of patient experiences and the complexity of their responses to dental anxiety, this study attempted to explore those factors through a mixed methods approach. A sample of 499 dentally anxious patients enrolled in a clinical trial and were interviewed about their degree of distress and avoidance of dental care. The goal of the RCT was to improve dental appointment attendance and reduce dental anxiety generally through the administration of an hour-long computerized intervention. The intervention was based on the principles of Cognitive Behavioral Therapy (CBT), and administered immediately preceding a patient's dental appointment. Previous studies examining the influence of CBT-based interventions on dental anxiety have consistently found at least a moderate reducing effect (14–16), suggesting that it may be a promising treatment for dental anxiety in both children and adult populations (15). This study posited that patients who used avoidant coping strategies, as well as those who could recall a prior negative dental experience, were more likely to experience higher levels of anxiety. While viewing the brief computerized intervention did lead to a long-term reduction in participant dental anxiety (17), the present study is a secondary analysis of the qualitative data collected during the clinical trial. Thus, this paper focuses on the patient experience as reported by participants.

## Materials and methods

### Procedures

This study was approved by the Temple University institutional review board (IRB, Protocol #24466). Through qualitative semi-structured interviews, the entire sample of 499 patients were asked how dental anxiety has impacted their lives. Questions asked by interviewers were open-ended, and participants were prompted to give details about their experiences. Researchers then explored what factors, such as dental anxiety origin and type of coping mechanisms, influenced the severity of dental anxiety or phobia. These interviews were conducted as part of a clinical trial to test the efficacy of a Cognitive Behavioral Intervention (CBI) to treat dental anxiety. While CBT is a form of psychotherapy consisting of manualized procedures that are administered in a therapeutic setting over the course of several sessions, a CBI is typically short-term and incorporates a limited number of CBT techniques. The present intervention consisted of an exposure hierarchy in which participants viewed their three most feared dental procedures in ascending order. For each of the three dental procedures selected, participants were provided information about what the procedure involves, shown examples of coping thoughts relevant to their selection, and then prompted to create their own coping thoughts while watching the procedure from a first-person perspective. As participants progressed through the intervention, they were prompted to rate their subjective units of distress in a gradual exposure paradigm.

Participants were randomized to one of three groups after baseline assessments: to view the CBI with a dentally trained staff member present in the room (*n* = 167), to view the same intervention but in the presence of psychology-trained personnel (*n* = 162), or to watch a time- and attention- matched control video (*n* = 170). Patients were asked to report what coping mechanisms they used during dental appointments at the baseline semi-structured interview, as well as one-month and three-months post intervention. Strategies used by dental patients across all arms of the study pre- and post- intervention were then compared. Questions regarding origin of dental anxiety were only asked at baseline, as anxiety origin was considered to be a stable characteristic that would be unaffected by the CBI.

### Participants

Patients with upcoming appointments at the Temple University School of Dentistry (TUKSoD) were invited to participate in this study. To be eligible, interested patients were required to either exceed a score of 18 on the Modified Dental Anxiety Scale (MDAS) or score a 4 or above on at least two items, as well as report a nonzero level of anxiety-related interference in their dental care on a 0–8 scale.

### Measures

The instruments administered in this study were chosen based on their previous use in studies of dental anxiety, as well as their internal consistency. Participants were assessed as follows:

Modified Dental Anxiety Scale (18, 19) (MDAS): The MDAS is a measure of dental anxiety severity consisting of 5 items. Respondents are asked to rate their fear of dental situations on a Likert scale from 1 (not anxious) to 5 (extremely anxious). These situations range in intensity from sitting in the waiting room of a dental clinic to having a local anesthetic injection above an upper back tooth. The total score ranges from 5 to 25, with scores exceeding 18 indicative of severe dental anxiety. The MDAS was chosen over other instruments like the Corah Dental Anxiety Scale (CDAS) (19) and the Dental Fear Maintenance Questionnaire (DFMQ) (20) due to its brevity, accessible language, and construct validity.

Pain Sensitivity Index (21) (PSI): The PSI is a 16-item measure which assesses the expected consequences of pain, as well as the expected severity of future pain. Items are rated on a scale from 0 (not at all) to 7 (very much), with higher scores indicating higher levels of pain sensitivity. In previous studies with dental patients, the PSI has demonstrated high internal consistency (*α* = 0.92).

Distress Tolerance Scale (22) (DTS): The DTS is a 15-item measure with items ranging on a scale from 1 (strongly agree) to 5 (strongly disagree) which measures the respondent's ability to tolerate distressing situations and emotional states. The total score ranges from 15 to 75, with higher scores indicating a higher level of tolerance to distress.

Fear Questionnaire Blood-Injury Injection Subscale (23) (FQBII): This is a subscale of the larger Fear Questionnaire featuring 5 items that specifically relate to the fear of seeing blood, injury, or receiving an injection. Items are rated from 0 (would not avoid it) to 8 (would always avoid it), with higher scores indicating a greater likelihood of avoidant behaviors common in phobias.

In addition, the Anxiety and Related Disorders Interview Schedule (24) (ADIS) was administered. The ADIS is a semi-structured interview designed to assess a patient's level of distress directly before and during a dental appointment. In addition to providing a template for qualitative data to be collected, the ADIS is distinct from the MDAS in that it also measures the level of interference dental anxiety has had on a respondent's dental care and daily life. The use of coping mechanisms is considered an indication of distress, as the number and type of coping mechanisms employed could be considered another measure of a participant's ability to tolerate dental procedures. After completing the ADIS, interviewers assign participants a clinical severity rating (CSR) based on their responses. A score of 4 or greater is considered within the range of clinical phobia, meaning that the dental anxiety has caused some level of avoidance of dental procedures in addition to distress.

Participants completed the ADIS and MDAS over the phone three times: first at baseline, and then at one- and three-months after their intervention and corresponding dental appointment. The PSI, DTS, and FQBII were also administered three times and completed independently by participants via Qualtrics, an online survey platform. All questions asked during the semi-structured interviews were the same across time points, with the exception of origin of dental anxiety, which was only assessed at baseline as part of the ADIS. Twenty percent of all baseline, one-month, and three-month ADIS recordings were reviewed and rated by another research assistant to ensure rating consistency. To maintain interviewer blindness to the assigned intervention condition, research assistants assigned as interventionists to one participant could not complete either the one-month or three-month ADIS for that same participant.

### Data management and analyses

ADIS interviews were audio recorded and interviewers took detailed notes throughout each session (debriefs). These debriefs were imported into NVivo, a qualitative data management software, for coding and thematic analyses. To establish a coding structure, the first 20 interview debriefs were independently coded by three members of the research team (i.e., triple coded) to identify and reach agreement on an initial set of codes. Once consensus was reached, an additional 20 interviews were triple-coded to confirm inter-coder reliability. Finally, the remaining interviews were double-coded. Coders met regularly to review and reach final coding decisions, with any discrepancies resolved through discussion with the third coder. Coders reviewed interview audio recordings as needed to clarify any ambiguity in the debriefs and to extract direct quotes from patient narratives.

Once the origins of dental anxiety and coping strategies were identified, they were further coded into subcategories (e.g., childhood vs. adulthood origin, positive vs. negative coping). Dummy codes were created to indicate whether participants endorsed a specific code or theme (e.g., origin of dental anxiety, use of coping mechanism). Dental anxiety origins and coping strategies were reported as frequencies and further examined for differences across intervention groups and assessment time-points. Specifically, participants endorsing coping strategies were compared for mean differences in dental anxiety severity (e.g., MDAS and CSR) and psychological factors (e.g., PSI and DTS). Planned analyses relied on bi-variate associations (e.g., chi-square and t-test), as the qualitative nature and low frequency of individual themes did not warrant more advanced analyses (e.g., repeated measures ANOVA). Coded qualitative data and quantitative data were analyzed using SPSS v28.0.

## Results

### Demographics

Participants were primarily female (70.6%) and reflected the broader demographics of patients at the dental school in North Philadelphia, with 62.4% reporting Black/African American race and an average age of 49 ± 14.9 years. Additional demographic data collected from the sample appears in Table 1.

**Table 1:** Demographics.

Age	Total (mean ± SD)	
Mean ± SD	499 (49.0 ± 14.9)	
Sex	*N*	%
Female	349	70.6
Male	140	28.3
Other	5	1
Race		
Asian	22	4.6
Black	301	62.4
Multiracial/other	30	6.3
White	129	26.8
Ethnicity		
Hispanic or Latino	56	11.4
Non-Hispanic	434	88.6

### Avoidant coping strategies

Thematic analysis revealed 30 distinct coping mechanisms used by participants either directly before or during their dental appointment, listed in full in Table 2. Of those coping mechanisms, the following avoidance strategies were identified: listening to music, trying to take mind off dental visit (such as through daydreaming), distractions on phone, reading, fidgeting/squeezing something, listening to music, playing a game, bringing a comforting object, sleeping during the appointment, and humming. These were classified as avoidance strategies based on their intended goal of diverting participant attention from their dental procedure.

**Table 2:** Reported coping mechanisms.

Code	Control			Dental			Psychology		
	*N* (%)^a^			*N* (%)^a^			*N* (%)^a^		
	Baseline	1 month	3 month	Baseline	1 month	3 month	Baseline	1 month	3 month
Anti-anxiety or other medication	10 (4.2)	9 (4.5)	11 (5.4)	12 (4.7)	7 (3.8)	3 (1.8)	11 (4.9)	6 (4.3)	5 (3.8)
Ask questions	2 (0.8)	2 (1.0)	6 (2.9)	4 (1.6)	6 (3.3)	2 (1.2)	3 (1.3)	4 (2.9)	1 (0.8)
Brings comforting object	0 (0)	0 (0)	0 (0)	1 (0.4)	0 (0)	0 (0)	1 (0.4)	1 (0.7)	0 (0)
Breathing techniques	37 (15.7)	28 (13.9)	32 (15.7)	26 (10.3)	21 (11.5)	22 (12.9)	30 (13.4)	15 (10.7)	16 (12.2)
Brings a friend	3 (1.3)	2 (1.0)	1 (0.5)	7 (2.8)	3 (1.6)	1 (0.6)	2 (0.9)	2 (1.4)	1 (0.8)
Cannabis or smoking	3 (1.3)	4 (2.0)	1 (0.5)	3 (1.2)	2 (1.1)	1 (0.6)	2 (0.9)	1 (0.7)	1 (0.8)
Closes eyes	8 (3.4)	3 (1.5)	6 (2.9)	5 (2.0)	2 (1.1)	3 (1.8)	7 (3.1)	1 (0.7)	2 (1.5)
Coping thoughts	6 (2.5)	7 (3.5)	2 (1.0)	7 (2.8)	22 (12.1)	14 (8.2)	5 (2.2)	10 (7.1)	11 (8.4)
Distractions on phone	9 (3.8)	7 (3.5)	9 (4.4)	10 (4.0)	4 (2.2)	6 (3.5)	15 (6.7)	4 (2.9)	6 (4.6)
Drinks water	2 (0.8)	2 (1.0)	0 (0)	2 (0.8)	3 (1.6)	1 (0.6)	1 (0.4)	1 (0.7)	0 (0)
Eats	3 (1.3)	1 (0.5)	1 (0.5)	1 (0.4)	2 (1.1)	1 (0.6)	2 (0.9)	0 (0)	0 (0)
Fidget/squeezing something	5 (2.1)	10 (5.0)	18 (8.8)	6 (2.4)	8 (4.4)	11 (6.4)	9 (4.0)	6 (4.3)	10 (7.6)
Hums	0 (0)	1 (0.5)	0 (0)	0 (0)	1 (1.1)	1 (0.6)	1 (0.4)	1 (0.7)	0 (0)
Hypervigilance	1 (0.4)	0 (0)	0 (0)	1 (0.4)	0 (0)	0 (0)	0 (0)	0 (0)	0 (0)
Listens to music	27 (11.4)	19 (9.5)	19 (9.3)	21 (8.3)	14 (7.7)	18 (10.5)	21 (9.4)	13 (9.3)	12 (9.2)
Mindfulness or meditation	9 (3.8)	10 (5.0)	8 (3.9)	9 (3.6)	6 (3.3)	6 (3.5)	7 (3.1)	5 (3.6)	5 (3.8)
No coping skills	17 (7.2)	14 (7.0)	9 (4.4)	20 (7.9)	10 (5.5)	6 (3.5)	16 (7.1)	10 (7.1)	7 (5.3)
Plays games	5 (2.1)	4 (2.0)	1 (0.5)	4 (1.6)	3 (1.6)	2 (1.2)	6 (2.7)	3 (2.1)	1 (0.8)
Prayer	14 (5.9)	10 (5.0)	15 (7.4)	18 (7.1)	9 (4.9)	6 (3.5)	16 (7.1)	6 (4.3)	6 (4.6)
Reading	4 (1.7)	2 (1.0)	3 (1.5)	9 (3.6)	3 (1.6)	4 (2.3)	10 (4.5)	3 (2.1)	0 (0)
Research procedures	2 (0.8)	3 (1.5)	4 (2.0)	5 (2.0)	1 (0.5)	0 (0)	5 (2.2)	4 (2.9)	1 (0.8)
Restricts eating	1 (0.4)	1 (0.5)	2 (1.0)	0 (0)	0 (0)	1 (0.6)	0 (0)	0 (0)	0 (0)
Routine before appointment	1 (0.4)	6 (3.0)	2 (1.0)	5 (2.0)	3 (1.6)	1 (0.6)	4 (1.8)	0 (0)	1 (0.8)
Self-talk	27 (11.4)	27 (13.4)	23 (11.3)	27 (10.7)	22 (12.1)	21 (12.3)	19 (8.5)	21 (15.0)	22 (16.8)
Sleep during apt	0 (0)	1 (0.5)	1 (0.5)	0 (0)	0 (0)	0 (0)	2 (0.9)	0 (0)	2 (1.5)
Starts conversations	8 (3.4)	3 (1.5)	5 (2.5)	12 (4.7)	5 (2.7)	10 (5.8)	5 (2.2)	7 (5.0)	2 (1.5)
Tell staff about anxiety or ask for breaks	7 (3.0)	1 (0.5)	8 (3.9)	7 (2.8)	6 (3.3)	3 (1.8)	5 (2.2)	1 (0.7)	0 (0)
Tries to take mind off dental visit	24 (10.2)	22 (10.9)	15 (7.4)	23 (9.1)	16 (8.8)	24 (14.0)	17 (7.6	15 (10.7)	17 (13.0)
Uses humor/tells jokes	1 (0.4)	1 (0.5)	2 (1.0)	3 (1.2)	1 (0.5)	1 (0.6)	2 (0.9)	0 (0)	0 (0)
Walks around	0 (0)	1 (0.5)	0 (0)	5 (2.0)	2 (1.1)	2 (1.2)	0 (0)	0 (0)	2 (1.5)
Total	236	201	204	253	182	171	224	140	131
Coping mechanism	Baseline			1 month			3 month		
	*N* (%)^a^			*N* (%)^a^			*N* (%)^a^		
	Intervention		Control	Intervention		Control	Intervention		Control
Avoidant^b^	125 (38.0)		63 (37.1)	75 (33.6)		55 (43.7)	74.0 (24,0)		54 (31.8)
Coping thoughts	12 (3.6)		6 (3.5)	32 (14.3)		7 (5.7)	25 (11.8)		2 (1.7)

^a^ Percentages reported are relative at each timepoint.

^b^ Includes distractions on phone, fidgets/squeezes something, hums, listens to music, plays games, reading, sleeps during apt, tries to take mind off dental visit.

### Origins of dental anxiety

The study also identified three themes of Dental Anxiety Origin: traumatic event in childhood, traumatic event in adulthood, and don't know (always anxious). For participants who could identify the appointment that first triggered their anxiety, the procedure they underwent, such as an extraction, root canal, or routine cleaning, was coded. Table 3 includes a full list of these themes.

**Table 3:** Reported origins of dental anxiety and themes.

Code	Baseline *N*	%
Source of dental anxiety		
Specific trauma		
Trauma in childhood	162	32.5
Trauma in adulthood	190	38.1
Fear of new procedures	23	4.6
Learned from others	30	6.0
Don't know (always anxious)	103	20.6
Thoughts and feelings		
Values	9	1.8
Trust in medical providers	84	16.8
Extreme event	35	7.0
Object in mouth	6	1.2
COVID	8	1.6
Fear of new provider	6	1.2
Progression of disease	53	10.6
Inadequate anesthetic	55	11.0
Different cultural experiences	4	0.8
Specific procedures		
Injection	68	13.6
Root canal	48	9.6
Drilling/cavity filling	53	10.6
Extraction	77	15.4
Implants	13	2.6
Other procedure	37	7.4

While reviewing the dataset of participant responses, researchers identified several frequently mentioned experiences that may contribute to a patient's dental anxiety. These are defined below with corresponding patient quotes:

*Loss of control:* When participants feel that they have no control over what happens to them while in the dental chair, their fear of the impending procedure intensifies.

“I've been in a situation before, not here, but with the dentist that left me with a constant fear. I got a tooth pulled and I felt something that I shouldn't have felt because I was supposed to be numb. So they ended up having to put me to sleep and waking up from that was not fun. But I think that reaction is to everybody or most people. I didn't like that feeling of being put out, losing control. I woke up in fear and crying.”

“I don't think anything led to it specifically, but I think literally like not having a whole lot of control in those situations makes me feel anxious.”

“I remind myself that it's going to happen and I'm going to feel some type of way. My life is in their hands and I have to just trust that they're not going to mess up or be negligent.”

*Trust in providers:* When patients believed they could no longer trust their providers, or that their dentist no longer had their best interest at heart, their anxiety increased.

“When I was 5 the man put the tongue depressor down to look at my throat and he jammed it. Another time the dentist came in and he looked high, when I was 16, and he just put his finger in my mouth and I'm a person who really likes clean hands and he hadn't washed his hands. And he messed my tooth up, one of the teeth that I've had trouble with. I've had to get a partial now because of what he did, too.”

“One time they said that I needed a root canal. Then when I went back they said they didn't say that. So I went to the specialist. They should have some kind of summary where they talk with the patient and say, ‘This is what you're going to need, this is the plan,’ and they don't do that. There should be some type of care plan or summary of what happened, what's the plan, and how much is it. And also I went to the dentist and had a real painful black and blue bruise on my cheek. So I called and said that it was from the camera that they use but really it was the injection. But he didn't say at the time that I might have a bruise. He waited until I called back asking and then he said it. If I was going to have a bruise he should have said, ‘You might have a bruise tomorrow.’ It's about being informed.”

*Fear of pain:* Patients reported worrying about the pain they would experience at the dentist, even as they acknowledged that their avoidance of the dentist contributed to painful oral health complications:

“I don't like to smile. My teeth are all messed up and I don't want people to see. Whatever I need to get done, I just want to be like a normal person after the dentist. I just want to be able to get this work done and go home..Just all the pulling and digging, it felt like it goes through my body when they start scraping and digging and all of those things. I don't like that. I really don't like that.”

“I recall one time when they took me I had an abscess on my gums and there was pus on my gums. They had to drain the pus out. That one minute right there was when I realized how scared I was, you know what I mean? I was a teenager, in my early teens, 14 maybe, and that's when it got started.”

“It was actually a time when I was hurting and I had opened those 800 Motrin ibuprofens because I was taking them back to back for so long for like a month straight and I had hurt my stomach and I had to go to the hospital when all that time I should have been going to the dentist because I was taking it for my tooth. So that's how much it has cost, like, interfered with my life. Like I'm so terrified of the dentist that I'm just like trying to avoid it as much as possible.”

### Mixed-methods analyses

There was no significant difference in participants who reported the use of coping thoughts similar to the strategies taught during the intervention at baseline, with 3.6% in the pooled intervention group and 3.5% in the control group endorsing these strategies (*X*^2^ = .004, *p* = .947). At one-month follow up, participants who received the cognitive behavioral intervention demonstrated an increase in the use of coping thoughts (14.3%), and were significantly more likely (*X*^2^ = 6.27, *p* = .012) to use this strategy when compared to the control group (5.7%).

Among those in the intervention group who endorsed the use of avoidant coping strategies, scores on both the MDAS and the FQBII were higher than the scores of participants who used no or non-avoidant strategies. Table 4 shows significant differences in both MDAS (F = 7.4, *p* < .001) and FQBII (F = .15, *p* < .018) were observed at one month. These differences remained present at three months post-intervention. The use of avoidant coping strategies was relatively equal between the intervention group (38%) and control group (37.1%) at baseline. However, a reduction in the use of avoidant coping strategies was observed among the intervention group (38.0% at baseline to 24.0% at three months), while the same effect was not observed in the control group (37.1% at baseline and 31.8% at three months). When comparing the intervention and control sample based on their use of avoidant coping mechanisms, the association between these coping mechanisms and mean differences in MDAS and FQBII remained significant in the intervention group, with higher scores on both measures reported by subjects who endorsed avoidant coping strategies. However, an independent samples t-test revealed that the association of avoidant coping strategies and the FQBII was no longer significant in the control group (*p* > .05).

**Table 4:** Associations among psychological factors and coping mechanisms at one month.

Measure	Full sample				Intervention				Control			
	Avoidant coping (M ± SD)	Other coping (M ± SD)	t	*p*	Avoidant coping (M ± SD)	Other coping (M ± SD)	t	*p*	Avoidant coping (M ± SD)	Other coping (M ± SD)	t	*p*
MDAS	18.6 ± 3.9	16.6 ± 4.8	−4.01	**<** **.** **001**	18.3 ± 3.7	16.3 ± 4.9	−2.99	**0** **.** **003**	19.0 ± 4.1	17.1 ± 4.4	−2.41	**0** **.** **017**
PSI	63.2 ± 23.9	60.6 ± 24.5	−0.97	0.331	62.0 ± 22.7	60.4 ± 24.5	−0.45	0.653	65.0 ± 25.5	60.9 ± 24.8	−0.88	3.83
ASI	27.4 ± 16.9	26 ± 16.2	−0.74	0.458	28.0 ± 16.7	25.1 ± 16.0	−1.20	0.232	26.6 ± 17.4	27.8 ± 16.7	0.39	0.695
DTS	46.8 ± 14.0	46.6 ± 13.9	−0.11	0.909	47.4 ± 12.9	46.7 ± 14.0	−3.77	0.707	45.8 ± 15.4	46.4 ± 13.7	0.20	0.841
FQBII	15.2 ± 10.1	12.5 ± 10.1	−2.37	**0** **.** **018**	16.1 ± 10.6	12.8 ± 9.3	−2.35	**0** **.** **019**	13.9 ± 9.3	11.7 ± 11.7	−1.09	0.278

Values in bold indicate statistical significance.

Of the three themes identified for Dental Anxiety Origin, the most commonly reported reason was a distressing dental procedure during adulthood (*n* = 190). Table 5 illustrates that significant differences in MDAS were observed (F = 9.4, *p* < .001) with subjects who reported a childhood trauma having the highest levels of dental anxiety (M = 20.8, SD = 2.8) relative to those who developed dental anxiety in adulthood (M = 19.2, SD = 3.7) or unknown origin (M = 19.9, SD = 3.3). The most reported dental procedures during a traumatic visit were: extraction (29%), injection (27%), drilling, cavity filling (20%), root canal (19%), and implants (5%). Patients further described perceived barriers to attending their appointments, as well as what caused them the most discomfort once they were in the dental chair. Reports of a lack of trust in medical providers were present in 84 of the 499 interviews conducted (16.8%). The perception of worsening disease resulting in more painful procedures, as well as receiving inadequate anesthetic during an appointment were also common in participant interviews and provided as reasons participants may be hesitant to seek out needed dental care.

**Table 5:** MDAS and CSR by dental anxiety origin.

Measure	Origin	Mean	*SD*	*F*	*p*
MDAS	Childhood	20.8	2.8	9.4	**<.001**
	Adulthood	19.2	3.7		
	Always/no specific reason	19.8	3.3		
CSR	Childhood	4.7	1.6	1.8	0.161
	Adulthood	4.3	1.7		
	Always/no specific reason	4.6	1.7		

Values in bold indicate statistical significance.

## Discussion

These results support the hypothesis that early onset of dental anxiety leads to greater anxiety severity, and further validates previous literature on avoidant coping strategies also demonstrating some association with higher anxiety (9). Following a brief CBI, the use of avoidant coping strategies did decrease, as well as the level of patient anxiety severity as measured by the MDAS. However, these results only capture a limited picture of the patient experience. Conducting semi-structured interviews allowed patients to describe their anxiety in their own words and contribute to a narrative of the most common issues experienced by patients during their dental care. Notably, the use of skills specifically taught during the intervention increased among participants in the intervention group, suggesting that some participants made use of the skills they had learned during the brief CBI even three months after their research visit. Interviewers did not prompt participants to identify these skills but instead recorded when they were spontaneously identified. Although brief, the intervention provided participants with examples of proactive coping mechanisms and was tailored specifically to reduce anxiety during an appointment, which may explain its sustained effects.

Patients frequently described experiences characterized by lack of control. The relationship between loss of control and symptoms of anxiety has previously been established in the literature, supporting the findings of the present study (25). Patients spoke openly about experiences where they felt they had very little control of their dental appointment, citing those experiences as reasons why their anxiety increased. Additionally, the present study, which did not ask participants directly about their relationship with their provider, nevertheless discovered themes of the importance of being able to trust one's dentist. This is also consistent with present literature. In one study exploring trust between patients and their primary care physicians, researchers found that patients with diabetes who endorsed a trusting relationship with their physician also had lower levels of anxiety and depression (26). In another study of adherence to 2020 pandemic guidelines, participants who reported higher levels of trust in the healthcare system were more likely to follow social distancing and risk reduction guidance (27).

Notably, the qualitative data collected for this study demonstrated narrative themes that are common among the low-income, majority Black/African American community which the dental school primarily serves; namely, mistrust of medical providers. Previous studies exploring institutional mistrust have found that African American and Hispanic patients are more likely to report low trust of healthcare professionals and less likely to utilize dental services (28, 29). Mistrust of participating in research is also common among marginalized communities due to historical experiences of exploitation and deception, leading to lower levels of representative research participation (30). The findings of this study reaffirm the long-term effects of healthcare inequities on the experience of marginalized community members who attempt to access dental care. By collecting a large sample of qualitative interviews from dentally anxious patients in a resource-limited community, this study intended to elevate the narrative voice of individuals who may be hesitant to utilize healthcare services or participate in research at all.

This study had several limitations. All participants interviewed were those with a dental home, meaning that those patients with the greatest severity of dental phobia were likely not recruited for this study. Additionally, participants who completed their baseline interview but did not attend their upcoming dental appointment were unlikely to reschedule their intervention visit. For those participants, coping strategies were only collected for their baseline assessment, although the data for dental anxiety origin is complete. This study is also limited in its generalizability to other populations, such as patients receiving care in non-urban environments or pediatric patients. Lastly, this study did not collect data encompassing a follow-up time period longer than three months.

Even so, these interviews contribute to the body of knowledge regarding a patient's reasons for pursuing or avoiding care. Additionally, themes of mistreatment and anticipation of pain were common reasons for developing anxiety, warranting greater sensitivity from dental practitioners who likely have patients who share in these experiences. Dental providers may also benefit from additional psychological education on dental anxiety, as this would provide additional context when treating anxious-presenting patients. Future qualitative studies on dental anxiety origins and coping mechanisms in other environments, such as at a rural dental school or traditional dental practice, would further broaden our understanding of dental anxiety development and may yield different findings.

## Data Availability

The raw data supporting the conclusions of this article will be made available by the authors, without undue reservation.
